# *catena*-Poly[[(dimethyl­formamide-κ*O*)copper(II)]-bis­(μ-4-nitro­phenyl­cyanamido-κ^2^ *N* ^1^:*N* ^3^)]

**DOI:** 10.1107/S160053680900796X

**Published:** 2009-03-11

**Authors:** Hossein Chiniforoshan, Soghra Jalilpour, Bahare Shirinfar, Hamid Reza Khavasi

**Affiliations:** a Department of Chemistry, Isfahan University of Technology, Isfahan 84156-38111, Iran; b Department of Chemistry, Shahid Beheshti University, G.C., Evin, Tehran 1983963113, Iran

## Abstract

In the title compound, [Cu(C_7_H_4_N_3_O_2_)_2_(C_3_H_7_NO)], the Cu^II^ atom is five-coordinated in a distorted square-pyramidal geometry, with the N atoms in equatorial positions and the dimethyl­formamide O atom in an axial position. The dihedral angle between adjacent benzene rings is 70.33 (12)°.

## Related literature

The phenyl­cyanamide molecule can function as bridging ligand and can coordinate to two different metallic centers by means of the nitrile and amine N atoms (μ_1,3_ bonding mode), forming di- and polynuclear complexes, see: Ainscough *et al.* (1991 ▶); Brader *et al.* (1990 ▶); Crutchley (2001 ▶); Escuer *et al.* (2004 ▶). For the magnetic properties of coordination polymers, see: Grosshenny *et al.* (1996 ▶). For the preparation of 4-NO_2_-phenyl­cyanamide used in the synthesis, see: Crutchley & Naklicki (1989 ▶).
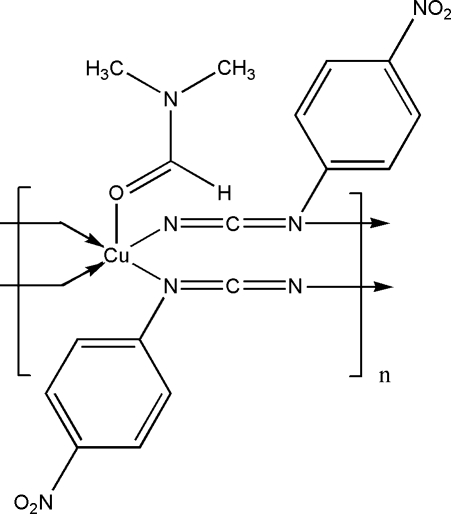


## Experimental

### 

#### Crystal data

[Cu(C_7_H_4_N_3_O_2_)_2_(C_3_H_7_NO)]
*M* *_r_* = 460.91
Monoclinic, 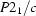

*a* = 21.5103 (12) Å
*b* = 8.7883 (5) Å
*c* = 9.9195 (5) Å
β = 101.746 (4)°
*V* = 1835.91 (17) Å^3^
*Z* = 4
Mo *K*α radiation
μ = 1.24 mm^−1^
*T* = 120 K
0.50 × 0.23 × 0.15 mm

#### Data collection

Stoe IPDS-II diffractometer
Absorption correction: numerical with shape of crystal determined optically *T*_min_ = 0.720, *T*_max_ = 0.832
13057 measured reflections
4874 independent reflections
4496 reflections with *I* > 2σ(*I*)
*R*_int_ = 0.050

#### Refinement

*R*[*F* ^2^ > 2σ(*F* ^2^)] = 0.033
*wR*(*F* ^2^) = 0.091
*S* = 1.08
4874 reflections
273 parameters
H-atom parameters constrained
Δρ_max_ = 0.99 e Å^−3^
Δρ_min_ = −0.91 e Å^−3^

### 

Data collection: *X-AREA* (Stoe & Cie, 2005 ▶); cell refinement: *X-AREA*; data reduction: *X-AREA*; program(s) used to solve structure: *SHELXTL* (Sheldrick, 2008 ▶); program(s) used to refine structure: *SHELXTL*; molecular graphics: *ORTEP-3 for Windows* (Farrugia, 1997 ▶); software used to prepare material for publication: *WinGX* (Farrugia, 1999 ▶).

## Supplementary Material

Crystal structure: contains datablocks global, I. DOI: 10.1107/S160053680900796X/bq2120sup1.cif

Structure factors: contains datablocks I. DOI: 10.1107/S160053680900796X/bq2120Isup2.hkl

Additional supplementary materials:  crystallographic information; 3D view; checkCIF report

## Figures and Tables

**Table:** 

N2—Cu1	2.0862 (13)
N5—Cu1	2.0599 (12)
Cu1—N4^i^	1.9648 (13)
Cu1—N1^ii^	1.9748 (13)
Cu1—O5	2.1443 (12)

**Table:** 

N4^i^—Cu1—N1^ii^	154.42 (6)
N4^i^—Cu1—N5	90.85 (5)
N1^ii^—Cu1—N2	90.41 (5)
N5—Cu1—N2	172.66 (5)
N4^i^—Cu1—O5	102.75 (5)
N1^ii^—Cu1—O5	102.83 (5)
N5—Cu1—O5	92.25 (5)
N2—Cu1—O5	95.08 (5)

Symmetry codes: (i) 
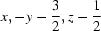
; (ii) 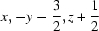.

**Fig. 1.**
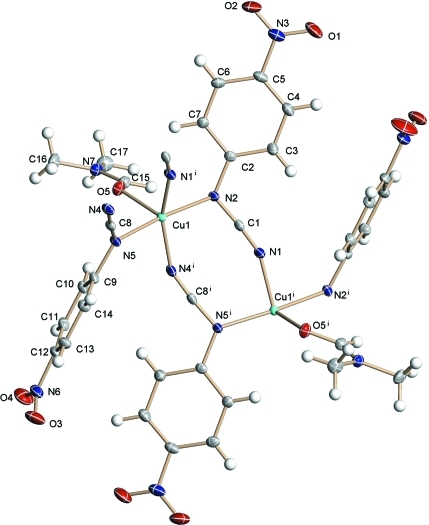
View of (I) with 30% probability displacement ellipsoids. Symmetry code (i): x, -y - 3/2, z - 1/2.

## supplementary crystallographic information

### Comment

Phenylcyanamide ligands are used in the construction of transition metal coordination complexes. The study of coordination polymeric materials holds great interest. The phenylcyanamide can function as bridging ligand and can coordinate to two different metallic centers by means of the nitrile and amine nitrogen (µ_1,3_ bonding mode), forming di- and polynuclear complexes (Brader *et al.*, 1990; Crutchley *et al.*, 2001; Ainscough *et al.*, 1991; Escuer *et al.*, 2004). It can modify the solubility and crystallinety of resulting compounds and there is the different coordination in complexes. We are attempting to construct conductive inorganic polymer chains that are cross-linked by cyanamide groups to a coordination complex. Coordination polymers also hold promise as novel materials because of their magnetic properties (Grosshenny *et al.*, 1996). More recently various aromatic cyanamide complexes have been studied by *x*-ray crystallography.

In the molecule of the title compound, (I), (Fig. 1) the selected bond lengths and angles are listed in Table 1. In this molecule, the {Cu(4—NO_2_-pcyd)_2_(DMF)}_n_ one-dimensional chain coordination polymer bridged by 4-NO_2_-phenylcyanamide. Each copper atom has a distored square pyramidal geometry, that nitrogen atoms are in equatorial position and oxygen atom from DMF molecule is in axial position (Table 1.). The dihedral angle between adjacent phenyl rings in the polymeric chain is 70.33 (12)°.

### Experimental

The 4-NO2-phenylcyanamide (Crutchley *et al.*,1989) (0.163 gr, 0.5 mmol) dissolved in methanol (30 ml) was added slowly to a solution of copper(II) acetate monohydrate (0.998 gr, 1 mmol) in methanol (30 ml). The mixture was stirred for 4 h. The solid filtered and crystals suitable for X-ray structure determination were obtained by dissolving in DMF then diffused by n-Hexane, after 1 week.

### Refinement

All of the H atoms were positioned geometrically, with C—H = 0.93 Å for aromatic and aldehyde H atoms and with C—H = 0.93 Å for methyl H atoms, and constrained to ride on their parent atoms, with *U*_iso_(H) = 1.2*U*_eq_(C).

### Crystal data

**Table:**

[Cu(C_7_H_4_N_3_O_2_)_2_(C_3_H_7_NO)]	*F*(000) = 940
*M**_r_* = 460.91	*D*_x_ = 1.668 Mg m^−^^3^
Monoclinic, *P*2_1_/*c*	Mo *K*α radiation, λ = 0.71073 Å
Hall symbol: -P 2ybc	Cell parameters from 1359 reflections
*a* = 21.5103 (12) Å	θ = 3.0–29.3°
*b* = 8.7883 (5) Å	µ = 1.24 mm^−^^1^
*c* = 9.9195 (5) Å	*T* = 120 K
β = 101.746 (4)°	Prism, violet
*V* = 1835.91 (17) Å^3^	0.5 × 0.23 × 0.15 mm
*Z* = 4	

### Data collection

**Table:**

Stoe IPDS-II diffractometer	4496 reflections with *I* > 2σ(*I*)
rotation method scans	*R*_int_ = 0.050
Absorption correction: numerical shape of crystal determined optically	θ_max_ = 29.3°, θ_min_ = 3.0°
*T*_min_ = 0.720, *T*_max_ = 0.832	*h* = −29→24
13057 measured reflections	*k* = −10→12
4874 independent reflections	*l* = −13→13

### Refinement

**Table:**

Refinement on *F*^2^	0 restraints
Least-squares matrix: full	H-atom parameters constrained
*R*[*F*^2^ > 2σ(*F*^2^)] = 0.033	*w* = 1/[σ^2^(*F*_o_^2^) + (0.051*P*)^2^ + 0.8628*P*] where *P* = (*F*_o_^2^ + 2*F*_c_^2^)/3
*wR*(*F*^2^) = 0.091	(Δ/σ)_max_ = 0.017
*S* = 1.08	Δρ_max_ = 0.99 e Å^−^^3^
4874 reflections	Δρ_min_ = −0.91 e Å^−^^3^
273 parameters	

### Special details

**Table:**

Geometry. All e.s.d.'s (except the e.s.d. in the dihedral angle between two l.s. planes) are estimated using the full covariance matrix. The cell e.s.d.'s are taken into account individually in the estimation of e.s.d.'s in distances, angles and torsion angles; correlations between e.s.d.'s in cell parameters are only used when they are defined by crystal symmetry. An approximate (isotropic) treatment of cell e.s.d.'s is used for estimating e.s.d.'s involving l.s. planes.

### Fractional atomic coordinates and isotropic or equivalent isotropic displacement parameters (Å^2^)

**Table:**

	*x*	*y*	*z*	*U*_iso_*/*U*_eq_	
C1	0.18470 (7)	−0.78476 (17)	0.48486 (14)	0.0134 (3)	
C2	0.11109 (7)	−0.85879 (17)	0.61210 (15)	0.0134 (3)	
C3	0.06225 (7)	−0.77637 (19)	0.52815 (15)	0.0170 (3)	
H3	0.0717	−0.7132	0.4599	0.02*	
C4	−0.00005 (7)	−0.78755 (19)	0.54535 (16)	0.0187 (3)	
H4	−0.0323	−0.7327	0.4893	0.022*	
C5	−0.01325 (7)	−0.8821 (2)	0.64786 (16)	0.0179 (3)	
C6	0.03397 (8)	−0.96827 (18)	0.73088 (16)	0.0177 (3)	
H6	0.0239	−1.0325	0.7978	0.021*	
C7	0.09602 (7)	−0.95722 (17)	0.71271 (15)	0.0155 (3)	
H7	0.1278	−1.015	0.7671	0.019*	
C8	0.31962 (7)	−0.74093 (18)	1.03158 (14)	0.0140 (3)	
C9	0.39374 (7)	−0.82569 (17)	0.90993 (15)	0.0142 (3)	
C10	0.41040 (7)	−0.94717 (18)	0.83254 (15)	0.0156 (3)	
H10	0.3799	−1.0178	0.7932	0.019*	
C11	0.47252 (7)	−0.96186 (19)	0.81480 (15)	0.0179 (3)	
H11	0.4839	−1.0419	0.7634	0.021*	
C12	0.51748 (8)	−0.8551 (2)	0.87497 (16)	0.0186 (3)	
C13	0.50288 (7)	−0.7369 (2)	0.95631 (16)	0.0192 (3)	
H13	0.534	−0.6688	0.9982	0.023*	
C14	0.44059 (7)	−0.72297 (19)	0.97349 (15)	0.0170 (3)	
H14	0.4299	−0.6448	1.0277	0.02*	
C15	0.23643 (8)	−1.18081 (18)	0.70530 (16)	0.0164 (3)	
H15	0.2162	−1.1469	0.6187	0.02*	
C16	0.27640 (9)	−1.3914 (2)	0.85538 (18)	0.0239 (3)	
H16A	0.2471	−1.4544	0.8914	0.029*	
H16B	0.2907	−1.3101	0.9187	0.029*	
H16C	0.3121	−1.4514	0.8431	0.029*	
C17	0.22428 (9)	−1.4360 (2)	0.61151 (18)	0.0242 (3)	
H17C	0.195	−1.5073	0.6375	0.029*	
H17B	0.2605	−1.4896	0.5928	0.029*	
H17A	0.2038	−1.382	0.5305	0.029*	
N1	0.19773 (6)	−0.73209 (16)	0.38708 (13)	0.0159 (2)	
N2	0.17467 (6)	−0.84608 (14)	0.59891 (13)	0.0133 (2)	
N3	−0.07813 (7)	−0.8905 (2)	0.67009 (16)	0.0245 (3)	
N4	0.30602 (6)	−0.68183 (17)	1.12639 (13)	0.0169 (3)	
N5	0.32972 (6)	−0.80866 (16)	0.92140 (12)	0.0136 (2)	
N6	0.58231 (7)	−0.8682 (2)	0.85134 (15)	0.0249 (3)	
N7	0.24485 (6)	−1.32833 (16)	0.72334 (14)	0.0166 (3)	
Cu1	0.252435 (8)	−0.842098 (19)	0.763487 (17)	0.01030 (7)	
O1	−0.11924 (6)	−0.8137 (2)	0.59671 (16)	0.0360 (3)	
O2	−0.08874 (8)	−0.9731 (2)	0.7622 (2)	0.0497 (5)	
O3	0.61904 (7)	−0.7620 (2)	0.88752 (16)	0.0380 (4)	
O4	0.59667 (7)	−0.9839 (2)	0.79478 (16)	0.0379 (4)	
O5	0.25389 (6)	−1.08324 (13)	0.79693 (11)	0.0182 (2)	

### Atomic displacement parameters (Å^2^)

**Table:**

	*U*^11^	*U*^22^	*U*^33^	*U*^12^	*U*^13^	*U*^23^
C1	0.0091 (6)	0.0152 (6)	0.0141 (6)	−0.0002 (5)	−0.0015 (5)	−0.0010 (5)
C2	0.0118 (6)	0.0143 (6)	0.0137 (6)	−0.0026 (5)	0.0017 (5)	−0.0023 (5)
C3	0.0150 (7)	0.0208 (7)	0.0146 (6)	0.0011 (5)	0.0017 (5)	0.0014 (5)
C4	0.0131 (7)	0.0228 (8)	0.0189 (6)	0.0024 (6)	0.0005 (5)	−0.0020 (6)
C5	0.0116 (6)	0.0208 (7)	0.0219 (7)	−0.0027 (6)	0.0047 (5)	−0.0059 (6)
C6	0.0162 (7)	0.0176 (7)	0.0204 (7)	−0.0046 (5)	0.0062 (5)	−0.0007 (5)
C7	0.0139 (6)	0.0158 (6)	0.0163 (6)	−0.0025 (5)	0.0019 (5)	0.0004 (5)
C8	0.0089 (6)	0.0183 (7)	0.0135 (6)	−0.0012 (5)	−0.0009 (5)	0.0010 (5)
C9	0.0115 (6)	0.0182 (7)	0.0125 (6)	0.0023 (5)	0.0015 (5)	0.0023 (5)
C10	0.0134 (6)	0.0177 (7)	0.0145 (6)	0.0020 (5)	0.0005 (5)	−0.0002 (5)
C11	0.0163 (7)	0.0224 (7)	0.0147 (6)	0.0060 (6)	0.0029 (5)	0.0012 (5)
C12	0.0116 (6)	0.0271 (8)	0.0173 (7)	0.0029 (6)	0.0035 (5)	0.0051 (6)
C13	0.0133 (6)	0.0246 (8)	0.0190 (7)	−0.0025 (6)	0.0019 (5)	0.0015 (6)
C14	0.0144 (7)	0.0204 (7)	0.0159 (6)	0.0002 (5)	0.0025 (5)	−0.0024 (5)
C15	0.0183 (7)	0.0142 (7)	0.0169 (6)	−0.0004 (5)	0.0039 (5)	0.0002 (5)
C16	0.0293 (8)	0.0163 (7)	0.0251 (8)	0.0047 (6)	0.0031 (6)	0.0053 (6)
C17	0.0303 (9)	0.0170 (7)	0.0265 (8)	−0.0018 (6)	0.0089 (7)	−0.0079 (6)
N1	0.0104 (5)	0.0230 (7)	0.0139 (5)	−0.0003 (5)	0.0014 (4)	0.0018 (5)
N2	0.0100 (5)	0.0181 (6)	0.0110 (5)	−0.0018 (4)	0.0006 (4)	0.0006 (4)
N3	0.0151 (6)	0.0283 (8)	0.0312 (7)	−0.0031 (6)	0.0074 (5)	−0.0066 (6)
N4	0.0105 (5)	0.0263 (7)	0.0130 (5)	−0.0018 (5)	−0.0001 (4)	−0.0031 (5)
N5	0.0093 (5)	0.0191 (6)	0.0119 (5)	0.0002 (4)	0.0007 (4)	−0.0023 (4)
N6	0.0147 (6)	0.0396 (9)	0.0211 (6)	0.0052 (6)	0.0049 (5)	0.0051 (6)
N7	0.0183 (6)	0.0130 (6)	0.0186 (6)	−0.0008 (4)	0.0039 (5)	−0.0012 (4)
Cu1	0.00955 (10)	0.01191 (10)	0.00912 (10)	−0.00041 (6)	0.00118 (6)	−0.00033 (5)
O1	0.0144 (6)	0.0544 (10)	0.0386 (8)	0.0052 (6)	0.0040 (5)	−0.0007 (7)
O2	0.0246 (7)	0.0595 (11)	0.0711 (12)	0.0005 (7)	0.0246 (8)	0.0261 (10)
O3	0.0161 (6)	0.0527 (10)	0.0463 (8)	−0.0059 (6)	0.0087 (6)	−0.0026 (7)
O4	0.0244 (7)	0.0487 (9)	0.0443 (8)	0.0094 (7)	0.0154 (6)	−0.0049 (7)
O5	0.0248 (6)	0.0119 (5)	0.0169 (5)	−0.0014 (4)	0.0017 (4)	−0.0005 (4)

### Geometric parameters (Å, °)

**Table:**

C1—N1	1.160 (2)	C13—C14	1.390 (2)
C1—N2	1.3099 (18)	C13—H13	0.93
C2—C3	1.402 (2)	C14—H14	0.93
C2—N2	1.4046 (19)	C15—O5	1.250 (2)
C2—C7	1.408 (2)	C15—N7	1.316 (2)
C3—C4	1.388 (2)	C15—H15	0.93
C3—H3	0.93	C16—N7	1.457 (2)
C4—C5	1.387 (2)	C16—H16A	0.96
C4—H4	0.93	C16—H16B	0.96
C5—C6	1.394 (2)	C16—H16C	0.96
C5—N3	1.458 (2)	C17—N7	1.457 (2)
C6—C7	1.386 (2)	C17—H17C	0.96
C6—H6	0.93	C17—H17B	0.96
C7—H7	0.93	C17—H17A	0.96
C8—N4	1.163 (2)	N1—Cu1^i^	1.9748 (13)
C8—N5	1.3010 (19)	N2—Cu1	2.0862 (13)
C9—C14	1.403 (2)	N3—O2	1.224 (2)
C9—C10	1.403 (2)	N3—O1	1.227 (2)
C9—N5	1.4126 (19)	N4—Cu1^ii^	1.9648 (13)
C10—C11	1.389 (2)	N5—Cu1	2.0599 (12)
C10—H10	0.93	N6—O3	1.229 (2)
C11—C12	1.392 (2)	N6—O4	1.230 (2)
C11—H11	0.93	Cu1—N4^i^	1.9648 (13)
C12—C13	1.390 (2)	Cu1—N1^ii^	1.9748 (13)
C12—N6	1.465 (2)	Cu1—O5	2.1443 (12)
			
N1—C1—N2	175.56 (15)	N7—C16—H16A	109.5
C3—C2—N2	121.98 (13)	N7—C16—H16B	109.5
C3—C2—C7	119.06 (14)	H16A—C16—H16B	109.5
N2—C2—C7	118.96 (13)	N7—C16—H16C	109.5
C4—C3—C2	121.05 (14)	H16A—C16—H16C	109.5
C4—C3—H3	119.5	H16B—C16—H16C	109.5
C2—C3—H3	119.5	N7—C17—H17C	109.5
C5—C4—C3	118.64 (14)	N7—C17—H17B	109.5
C5—C4—H4	120.7	H17C—C17—H17B	109.5
C3—C4—H4	120.7	N7—C17—H17A	109.5
C4—C5—C6	121.70 (14)	H17C—C17—H17A	109.5
C4—C5—N3	119.07 (15)	H17B—C17—H17A	109.5
C6—C5—N3	119.22 (15)	C1—N1—Cu1^i^	158.01 (12)
C7—C6—C5	119.36 (14)	C1—N2—C2	116.60 (12)
C7—C6—H6	120.3	C1—N2—Cu1	114.97 (10)
C5—C6—H6	120.3	C2—N2—Cu1	124.69 (9)
C6—C7—C2	120.15 (14)	O2—N3—O1	123.22 (16)
C6—C7—H7	119.9	O2—N3—C5	118.16 (16)
C2—C7—H7	119.9	O1—N3—C5	118.63 (16)
N4—C8—N5	175.15 (15)	C8—N4—Cu1^ii^	152.54 (13)
C14—C9—C10	119.68 (14)	C8—N5—C9	116.64 (12)
C14—C9—N5	121.22 (13)	C8—N5—Cu1	117.20 (10)
C10—C9—N5	119.09 (14)	C9—N5—Cu1	124.94 (9)
C11—C10—C9	119.96 (15)	O3—N6—O4	123.70 (16)
C11—C10—H10	120	O3—N6—C12	118.03 (16)
C9—C10—H10	120	O4—N6—C12	118.26 (16)
C10—C11—C12	119.05 (14)	C15—N7—C17	121.62 (15)
C10—C11—H11	120.5	C15—N7—C16	121.55 (14)
C12—C11—H11	120.5	C17—N7—C16	116.82 (14)
C13—C12—C11	122.23 (14)	N4^i^—Cu1—N1^ii^	154.42 (6)
C13—C12—N6	119.11 (15)	N4^i^—Cu1—N5	90.85 (5)
C11—C12—N6	118.65 (15)	N1^ii^—Cu1—N5	88.36 (5)
C14—C13—C12	118.31 (15)	N4^i^—Cu1—N2	87.13 (5)
C14—C13—H13	120.8	N1^ii^—Cu1—N2	90.41 (5)
C12—C13—H13	120.8	N5—Cu1—N2	172.66 (5)
C13—C14—C9	120.69 (14)	N4^i^—Cu1—O5	102.75 (5)
C13—C14—H14	119.7	N1^ii^—Cu1—O5	102.83 (5)
C9—C14—H14	119.7	N5—Cu1—O5	92.25 (5)
O5—C15—N7	124.33 (15)	N2—Cu1—O5	95.08 (5)
O5—C15—H15	117.8	C15—O5—Cu1	124.92 (11)
N7—C15—H15	117.8		
			
N2—C2—C3—C4	−178.27 (14)	C14—C9—N5—C8	−27.7 (2)
C7—C2—C3—C4	1.7 (2)	C10—C9—N5—C8	152.76 (14)
C2—C3—C4—C5	0.1 (2)	C14—C9—N5—Cu1	139.25 (12)
C3—C4—C5—C6	−1.6 (2)	C10—C9—N5—Cu1	−40.26 (19)
C3—C4—C5—N3	177.78 (15)	C13—C12—N6—O3	−10.4 (2)
C4—C5—C6—C7	1.3 (2)	C11—C12—N6—O3	169.52 (16)
N3—C5—C6—C7	−178.12 (14)	C13—C12—N6—O4	170.58 (16)
C5—C6—C7—C2	0.6 (2)	C11—C12—N6—O4	−9.5 (2)
C3—C2—C7—C6	−2.0 (2)	O5—C15—N7—C17	−178.97 (15)
N2—C2—C7—C6	177.94 (14)	O5—C15—N7—C16	−0.3 (3)
C14—C9—C10—C11	−2.5 (2)	C8—N5—Cu1—N4^i^	147.21 (12)
N5—C9—C10—C11	177.06 (13)	C9—N5—Cu1—N4^i^	−19.70 (13)
C9—C10—C11—C12	0.2 (2)	C8—N5—Cu1—N1^ii^	−7.22 (12)
C10—C11—C12—C13	2.2 (2)	C9—N5—Cu1—N1^ii^	−174.13 (13)
C10—C11—C12—N6	−177.70 (14)	C8—N5—Cu1—O5	−110.00 (12)
C11—C12—C13—C14	−2.2 (2)	C9—N5—Cu1—O5	83.09 (13)
N6—C12—C13—C14	177.69 (14)	C1—N2—Cu1—N4^i^	−21.30 (11)
C12—C13—C14—C9	−0.1 (2)	C2—N2—Cu1—N4^i^	−178.68 (12)
C10—C9—C14—C13	2.5 (2)	C1—N2—Cu1—N1^ii^	133.23 (11)
N5—C9—C14—C13	−177.06 (14)	C2—N2—Cu1—N1^ii^	−24.15 (12)
C3—C2—N2—C1	−16.4 (2)	C1—N2—Cu1—O5	−123.85 (11)
C7—C2—N2—C1	163.60 (14)	C2—N2—Cu1—O5	78.76 (12)
C3—C2—N2—Cu1	140.62 (12)	N7—C15—O5—Cu1	171.10 (11)
C7—C2—N2—Cu1	−39.34 (18)	N4^i^—Cu1—O5—C15	−57.95 (14)
C4—C5—N3—O2	−179.12 (18)	N1^ii^—Cu1—O5—C15	121.84 (13)
C6—C5—N3—O2	0.3 (3)	N5—Cu1—O5—C15	−149.33 (13)
C4—C5—N3—O1	0.3 (2)	N2—Cu1—O5—C15	30.25 (13)
C6—C5—N3—O1	179.73 (16)		

Symmetry codes: (i) *x*, −*y*−3/2, *z*−1/2; (ii) *x*, −*y*−3/2, *z*+1/2.
